# Predisposing Factors for Sexual Dysfunction in Multiple Sclerosis

**DOI:** 10.3389/fneur.2021.618370

**Published:** 2021-02-09

**Authors:** Patrick Altmann, Fritz Leutmezer, Katharina Leithner, Tobias Monschein, Markus Ponleitner, Miranda Stattmann, Paulus Stefan Rommer, Tobias Zrzavy, Gudrun Zulehner, Klaus Berek, Thomas Berger, Gabriel Bsteh

**Affiliations:** ^1^Department of Neurology, Medical University of Vienna, Vienna, Austria; ^2^Department of Psychoanalysis and Psychotherapy, Medical University of Vienna, Vienna, Austria; ^3^Department of Neurology, Medical University of Innsbruck, Innsbruck, Austria

**Keywords:** sexuality, sexual dysfunction–risk factors, multiple sclerosis, patient reported outcome, risk

## Abstract

Sexual dysfunction (SD) in people with multiple sclerosis (pwMS) has a detrimental impact on individual health-related quality of life (HRQoL). It is not clear whether SD in multiple sclerosis (MS) is an independent symptom or merely a byproduct of other symptoms such as depression or anxiety. This cross-sectional study of 93 pwMS determines risk factors for SD in MS based on prevalence, HRQoL, and associated disease outcomes. Diagnosis of SD was determined based on the Multiple Sclerosis Intimacy and Sexuality Questionnaire-19 (MSISQ-19) and correlated with physical disability (measured by Expanded Disability Status scale, EDSS), depression and anxiety [Hospital Anxiety and Depression Scale (HADS)], and HRQoL [Multiple Sclerosis Quality of Life-54 (MSQoL-54)]. Multivariate regression models were performed to determine independent risk factors for SD in pwMS. Almost half of the participants in this study (46%) reported SD. HRQoL was significantly poorer in patients with MS suffering from SD (median [IQR] MSQoL-54 scores: physical subscale 52 [41–68] vs. 81 [69–89], *p* < 0.001; mental subscale 50 [38–82] vs. 86 [70–89], *p* < 0.001). In the multivariate model, EDSS was the only independent risk factor for SD (OR 18.1 for EDSS ≥4 [95% CI 3.3–31.4, *p* < 0.001]), while depression and anxiety were not. We conclude that the risk for SD is growing with increasing EDSS and is independent of depression or anxiety. Screening for SD becomes particularly relevant in patients with growing disability.

## Introduction

Sexual dysfunction (SD) is perceived to be more common in multiple sclerosis (MS) than in the general population. Studies report a prevalence of 50–90% in men and 40–80% in women (1–4). In context with SD in pwMS stemming from a multifactorial etiology, it is poorly understood whether SD is an independent symptom or a byproduct of other symptoms such as depression or anxiety. Furthermore, it remains to be elucidated whether there is a particular subgroup of pwMS at higher risk for SD and, thus, should be screened for SD.

Symptoms of SD may occur as a direct consequence of demyelinating lesions and their location in the central nervous system. Psychosocial domains also play an important role (5). Therefore, a division of SD into three components has been suggested. In short, primary SD occurs when neurologic pathways responsible for sensation or sexual response are affected. Secondary SD entails indirect changes in sexual response due to MS symptoms, e.g., fatigue or spasticity, and tertiary SD involves the biopsychosocial burden of MS on the individual and surfaces as a feeling of being less attractive, mood disorders, or fear of sexual rejection (6–8).

It is known that MS disability in general lowers a patient's health-related quality of life (HRQoL) significantly and SD in particular can add to that effect (9, 10). One study even suggests that SD has a larger negative influence on health-related quality of life than physical disability alone (11). Nonetheless, SD remains substantially underdiagnosed in people with MS for various reasons (3). Furthermore, communication about sexuality is not part of routine care, and there is certainly a need for further education and interdisciplinary care (12). Up to 90% of pwMS reported to have never discussed their sexuality with their treating neurologist (2, 13, 14). Therefore, knowing about possible risk factors for SD in pwMS may help in identifying patients who would particularly benefit from screening for SD.

In the present study, we investigated the prevalence of SD in a representative cohort of pwMS and compared disease characteristics and patient-reported outcome measures (PROMs) in patients with and without SD in order to identify independent risk factors for SD.

## Materials and Methods

### Ethics and Consent

The ethics committee at the Medical University of Vienna, Austria approved this study (EK1967/2018). Written informed consent was obtained from each patient, and we followed the guidelines set by the Declaration of Helsinki. We followed STROBE guidelines in this report (15).

### Study Population

We recruited 100 pwMS from our MS outpatient department from April 2019 through March 2020 according to current McDonald criteria (16). In total, we asked 114 patients to participate. Reasons given for not wanting to participate were: (i) lack of time to fill out the questionnaires, (ii) not being interested in participating in a research study, and (iii) not being interested in disclosing their sexuality. Treating neurologists documented clinical characteristics of participating patients including age and sex, disease duration in years, disease phenotype [relapsing MS, progressive MS (17)], Expanded Disability Status Scale [EDSS (18)], number of relapses over the past 12 months, presence of any bladder or bowel dysfunction (yes or no), presence of any sensory disturbance (yes or no), and disease-modifying treatment [DMT, categorized as moderately effective (dimethyl fumarate, glatiramer acetate, interferons, teriflunomide), highly active treatment (cladribine, fingolimod, natalizumab, ocrelizumab, rituximab)], or no treatment. Participants were asked to report on family status (single, relationship, married), number of children, and education (≤9 years of schooling, secondary schooling (highschool and equivalent), and college/university degree).

### Patient-Reported Outcome Measures

In order to investigate the three components of sexual functioning, MS-related quality of life, and depression or anxiety, each study participant completed three validated questionnaires. The Multiple Sclerosis Intimacy and Sexuality Questionnaire-19 (MSISQ-19) is a validated tool to report on sexual (dys-)function in people with MS (19, 20). It is composed of 19 questions gauging the occurrence of several sexual symptoms over the past 6 months. Patients can attribute scores ranging from 1 (symptom never occurs) to 5 (constant symptoms). The MSISQ-19 is able to capture three different components of sexual dysfunction (SD): primary, secondary, and tertiary (21). However, these distinctions are not mutually exclusive; therefore, patients can score positive in either combination of components. Overall, study participants were classified as having SD if they tested positive in at least one domain.

The Multiple Sclerosis Quality of Life-54 (MSQoL-54) questionnaire is derived from the Short-Form (36-item) Health Survey (SF-36) and provides a comprehensive assessment of MS-related quality of life from the patient's perspective (22). It generates two composite scores: the physical composite score (PCS) and the mental composite score (MCS) each ranging from 0 (poor HR-QoL) to 100 (best HR-QoL).

The Hospital Anxiety and Depression Scale (HADS) includes 14 items, seven for depression and anxiety each (23). HADS is a reliable and validated screening tool with reasonable psychometric properties for pwMS. Total scores for both subscales range from 0 (not affected) to 21 (most affected). Two different cut-offs have been suggested to distinguish unaffected cases from borderline and manifest ones (24, 25). In our study, we used the conservative cut-off of eight or higher.

### Statistical Analyses

Statistical analyses were performed using SPSS 25.0 (SPSS Inc, Chicago, IL, USA). Categorical variables were expressed in frequencies and percentages, continuous variables were tested for normal distribution by the Kolmogorov-Smirnov test and displayed as mean and standard deviation (StD) or median and interquartile range (IQR) as appropriate. Bivariate comparisons for categorical variables were calculated using the Chi-square test. For continuous variables, the independent *t* test or Mann-Whitney *U* test were applied as appropriate. Due to the exploratory nature of this study, formal *a priori* power and sample size calculation was not feasible, and we elected to forgo correction for multiple testing. Thus, estimation of effect sizes is arbitrary. Univariate and multivariate regression models were performed to calculate the odds ratios (OR) with 95% confidence intervals (95% CI) for SD based on anxiety, depression, and EDSS ≥4. To investigate risk factors for SD, we first performed univariate binary logistic regression models to identify those variables associated with SD. Those variables with a *p*-value <0.2 entered a multivariate regression model with SD as the dependent variable corrected for age and sex.

## Results

### Patient Characteristics and Sociodemographic Information

Out of 100 patients participating in this study, we report results from 93 patients whose information was complete and used for final analysis. The mean age of our cohort was 39 years (StD, 11.4), 19% were above 50 years old, and sex distribution was 3:2 (f:m). At the time of enrollment, two-thirds of patients had relapsing MS. The median EDSS was 2.0 (IQR, 0–4.5) with one-third of patients being rated as 4.0 or higher. Table 1 lists clinical characteristics examined in this study.

**Table 1:** Sociodemographic and clinical characteristics of patients.

	**Category**	**Value**
Participants analyzed	Number	93
Age^†^	Overall age	39.3 (11.4)^‡^
	18–35	36 (39%)
	36–49	39 (42%)
	>50	18 (19%)
Sex^‡^	Female	53 (57%)
	Male	40 (43%)
Disease phenotype^†^	Relapsing MS	65 (70%)
	Progressive MS	28 (30%)
EDSS^†^	Median	2 (0–4.5)^§^
	0–3.5	63 (68%)
	4 or above	30 (32%)
Number of relapses^‡^	Last 12 months	0.46 (0,8)
Disease duration^‡^	Years	8.2 (6.7)
Bladder or bowel dysfunction^†^	Yes	33 (35%)
Sensory dysfunction^†^	Yes	37 (40%)
MS medication^†^	Moderately effective	31 (33%)
	Highly active	47 (51%)
	No treatment	15 (16%)
Family status^†^	Single	29 (31%)
	Relationship	31 (33%)
	Married	33 (35%)
Children^†^	0	56 (60%)
	1	12 (13%)
	2	23 (25%)
	3	2 (2%)
Education^†^	≤9 years of schooling	36 (39%)
	Secondary schooling	28 (30%)
	College degree	29 (31%)

† *Absolute number (%)*.

‡ *Mean [standard deviation (StD)]*.

§ *Median [interquartile range (IQR)]*.

*EDSS, Expanded Disability Status Scale; MS, multiple sclerosis*.

### Prevalence of Sexual Dysfunction, Anxiety, and Depression

We found that 46% (*n* = 43) of the patients included in this study reported sexual dysfunction according to MSISQ-19 criteria. From patients experiencing SD, 37% were classified as experiencing secondary SD, 29% primary SD, and 19% tertiary SD (Table 2). The majority of participants with SD scored positive in only one domain (44%). Based on the HADS, we discovered that 34% scored positive for anxiety and 16% for depression. Median overall scores on the MSQoL-54 were 70 (IQR, 51–85) on the physical subscale and 76 (IQR, 50–88) on the mental subscale.

**Table 2:** Patient reported outcome measures (whole population).

	**Value**
Participants analyzed	93 (100%)
MSISQ-19: unaffected^†^	50 (54%)
MSISQ-19: primary sexual dysfunction^†^	27 (29%)
MSISQ-19: secondary sexual dysfunction^†^	34 (37%)
MSISQ-19: tertiary sexual dysfunction^†^	18 (19%)
MSISQ-19: positive in one domain^†^	19 (44%)
MSISQ-19: positive in two domains^†^	12 (28%)
MSISQ-19: positive in all three domains^†^	12 (28%)
HADS: anxiety^†^	32 (34%)
HADS: depression^†^	15 (16%)
MSQoL-54: physical^‡^	70 (51–85)
MSQoL-54: mental^‡^	76 (50–88)

† *Absolute number (%)*.

‡ *Median [interquartile range (IQR)]*.

*HADS, Hospital Anxiety and Depression Scale; MSISQ-19, Multiple Sclerosis Intimacy and Sexuality Questionnaire-19; MSQoL-54, Multiple Sclerosis Quality of Life-54 questionnaire*.

### Comparison of Patients With and Without Sexual Dysfunction

Clinical parameters of patients with and without SD are shown in Table 3. Patients with SD were more likely to have progressive MS (44 vs. 18%, *p* = 0.006), had a median EDSS of 4.0 or higher (56 vs. 12%, *p* < 0.001), and with bladder or bowel dysfunction (61 vs. 16%, *p* < 0.001). Concerning PROMs, we found participants with SD more likely to be depressed (28 vs. 6%, *p* = 0.005), whereas anxiety was distributed equally in both groups. Furthermore, quality of life was significantly poorer in pwMS and SD (MSQoL-54 physical composite 52 vs. 81, *p* < 0.001 and MSQoL-54 mental composite 50 vs. 86, *p* < 0.001). We found no significant differences concerning age, sex, disease duration, number of relapses within the past year, presence of sensory dysfunction, MS medication, and social information (family status, number of children, education).

**Table 3:** Associations between disease characteristic sexual dysfunction.

	**Category**	**No sexual dysfunction^†^**	**Any sexual dysfunction^†^**	***p*-value**
Participants analyzed^†^	Number	50 (54%)	43 (46%)	N/A
HADS: anxiety^†^	Score	15 (30%)	17 (40%)	n.s.
HADS: depression^†^	Score	3 (6%)	12 (28%)	0.005
MSQoL-54: physical^‡^	Score	81 (69–89)	52 (41–68)	<0.001
MSQoL-54: mental^‡^	Score	86 (70–89)	50 (38–82)	<0.001
Age^†^	Overall age	37^**‡**^ (29–46)	40 (34–50)	n.s.
	18–35	24 (48%)	12 (28%)	n.s.
	36–49	19 (38%)	20 (47%)	
	>50	7 (14%)	11 (25%)	
Sex^†^	Female	28 (56%)	25 (58%)	n.s.
	Male	22 (44%)	18 (42%)	
Disease phenotype^†^	Relapsing MS	41 (82%)	24 (56%)	0.006
	Progressive MS	9 (18%)	19 (44%)	
EDSS^†^	Median	1^**‡**^ (0–3)	4 (1.5–6)	<0.001
	0–3.5	44 (88%)	19 (44%)	<0.001
	4 or above	6 (12%)	24 (56%)	
Number of relapses^‡^	Last 12 months	0 (0–1)	0 (0–1)	n.s.
Disease duration^‡^	Years	6 (2–12)	8 (3–14)	n.s.
Bladder or bowel dysfunction^†^	Yes	8 (16%)	26 (61%)	<0.001
Sensory dysfunction^†^	Yes	16 (32%)	20 (47%)	n.s.
Family status^†^	Single	15 (30%)	14 (33%)	N/A
	Relationship	24 (48%)	7 (16%)	
	Married	11 (22%)	22 (51%)	
Children^‡^	Yes	0 (0-1)	0 (0–2)	N/A
Education^†^	≤9 years of schooling	18 (36%)	18 (42%)	N/A
	Secondary schooling	16 (32%)	12 (28%)	
	College degree	16 (32%)	13 (30%)	
MS medication^†^	Moderately effective	19 (38%)	12 (28%)	N/A
	Highly active	18 (36%)	29 (67%)	
	No treatment	13 (26%)	2 (5%)	

† *Absolute number (%)*.

‡ *Median [interquartile range (IQR)]*.

† *According to MSISQ-19*.

*HADS, Hospital Anxiety and Depression Scale; MSISQ-19, Multiple Sclerosis Intimacy and Sexuality Questionnaire-19; MS, multiple sclerosis; MSQoL-54, Multiple Sclerosis Quality of Life-54 questionnaire; N/A, not applicable or not performed; n.s., not significant*.

### Definition of Risk Factors for Sexual Dysfunction

**Figure 1:**
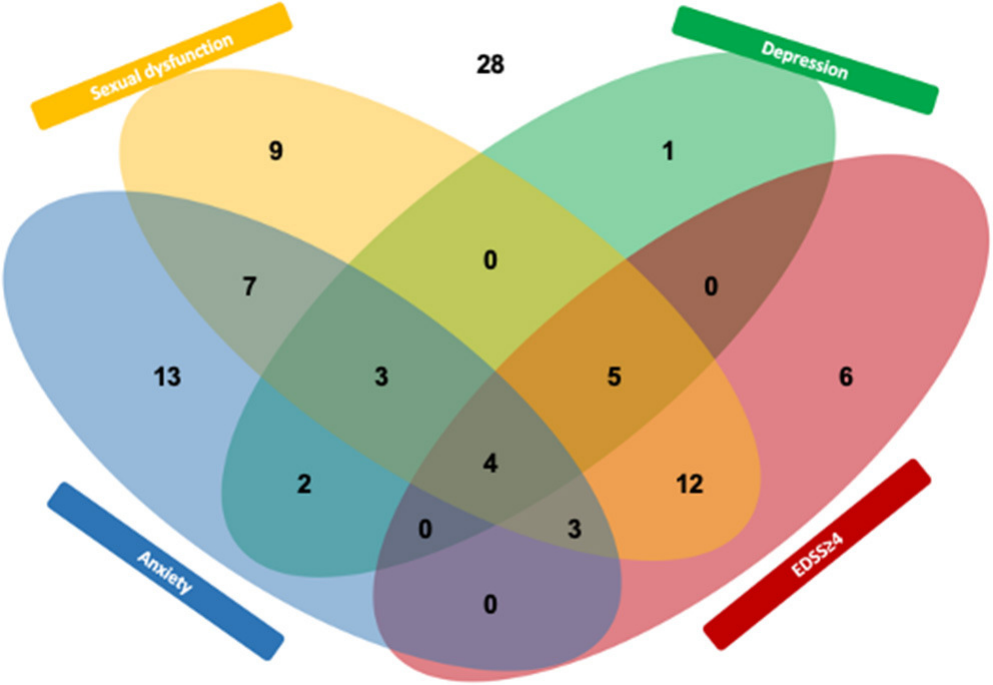
Interplay (Venn diagram) between sexual dysfunction and the MS outcome depression, anxiety, and EDSS ≥4. Numbers indicate participants from this study (*n* = 93).

Characterizing patient-related risk factors for SD, the multivariate model revealed EDSS ≥4 as strong risk factor for sexual dysfunction [OR, 18.1 (95% CI, 3.3–31.4), *p* < 0.001, Table 4]. While depression was univariately associated with SD [OR, 6.1 (95% CI, 1.6–23.3), *p* = 0.005], statistical significance was lost in the multivariate model [OR, 4.6 (95% CI, 0.9–23.2), *p* = 0.069]. Anxiety was not significantly associated with SD in either model. The interacting prevalence of SD, physical disability, depression, and anxiety is depicted in a Venn diagram (Figure 1).

**Table 4:** Risk factors for sexual dysfunction in multiple sclerosis.

	**OR**	**95% CI**	***p*-value**
Anxiety	2.2	0.7–6.8	0.159
Depression	4.6	0.9–23.2	0.069
EDSS ≥4	18.1	3.3–31.4	<0.001
	Nagelkerke *R*^2^ 0.366		

*Calculated by binary logistic regression model adjusted for age and sex*.

*EDSS, Expanded Disability Status Scale; OR, odds ratio; 95% CI, 95% confidence interval*.

## Discussion

There is a discrepancy between high prevalence rates for SD being reported in pwMS on one side and sexuality actually being addressed in clinical routine on the other. The aim of this study was to describe the risk of SD in order to characterize patients who may benefit from discussing their sexual history. In a representative cohort of pwMS, the prevalence of SD was 58% for women and 42% for men, comparing well with observations from other studies (2). SD was more common with increasing EDSS and resulted in poorer MS-related quality of life. Patients with SD were also significantly more likely to show symptoms of depression but not anxiety. When analyzing these factors combined in a multivariate model, EDSS was the only predictor of SD showing an 18-fold risk increase with an EDSS ≥4. In contrast, neither depression nor anxiety were independent predictors of SD.

Prevalence rates in earlier studies on sexual dysfunction in pwMS differed widely mainly due to use of measures that were not specific to MS. A strength of our study was the use of the MSISQ-19 which has been validated for the population of pwMS. With respect to studies using the MSISQ-19 to screen for SD, our study yielded a similar prevalence (19, 21, 26, 27). In this context, it is important to note how patients may be affected in more than one domain of the MSISQ-19. This distinction can be helpful for the management of SD in pwMS (28). Furthermore, the prevalence of anxiety and depression in our cohort can be considered representative as it is within the range reported in MS (29). While the rate of anxiety and depression in our sample was higher in univariate analyses in patients with SD compared with those without, EDSS emerged as the only *independent* risk factor for SD. It was somewhat surprising that neither depression nor anxiety emerged as (multivariate) risk factors for SD, as associations between them have been suggested (10, 26, 30). Even so, none of the studies reporting associations between SD and PROMs in pwMS performed multivariate analyses. This might explain why depression, which shows high MS-related prevalence to begin with, has been discussed as a prominent (univariate) risk factor. It seems that depression and anxiety are epiphenomena of increasing disability and sexual dysfunction which may have implications for understanding the burden of SD as being linked to physical disability. However, the cross-sectional design of our study cannot address the chicken-and-egg problem of whether depression and anxiety are additional causes of SD or a burdening consequence of disability. This would have to be investigated in a longitudinal study of patients beginning in early disease stages.

This study has some limitations. Our population was drawn from a single tertiary care center. Despite the fact that patients were treated by different neurologists, this still may have introduced bias in patient-reported outcomes. Furthermore, this study was cross-sectional and represents only one point in time. It would be interesting to know how the perception of sexuality changes within the individual patient over time. One study found that the prevalence of depression remained stable over 4 years while EDSS steadily increased (31). Additionally, this was an exploratory study without a formal *a priori* power and sample size calculation. That being said, a higher sample size would have allowed to perform a deeper analysis with multiple corrections and the estimation of effect size remains somewhat arbitrary. Moreover, when interpreting our findings, one must not forget that our conclusions emerge solely from the disease characteristics and PROMs chosen for this particular study. Disability measured on the Multiple Sclerosis Severity Scale (MSSS) for instance, may lead to different results, as it correlates better with HRQoL for some patients (32, 33). Nevertheless, we believe our results stress the importance of paying attention to this fairly underrepresented symptom and endeavors to implement a framework for the discussion of sexuality as part of clinical care are definitely warranted.

Barriers to communication about a patient's sexuality need to be acknowledged and lowered. The importance of addressing SD in clinical practice is apparent as prevalence rates estimated in studies and actual diagnoses of SD clearly oppose one another. Studies show only 2–6% of female and 6–10% of male pwMS have discussed their sexuality and sexual issues with their doctor or were actually diagnosed with SD. The reasons for this seem to surround elements of communication and education (1, 2, 9). It has been demonstrated that patients in general expect little help from their physicians on sex, giving reasons such as concerns of being dismissed by their doctor or lack of treatment options (34). Ultimately, it has been shown that simply talking about sexuality and sexual issues is highly beneficial. One study reported that 83% of women interviewed on their sexuality regarded it a positive experience (35). Similarly, the provision of educational material on SD alone can improve symptoms (36). Thus, every effort to raise awareness about this topic may not only enhance HRQoL in patients affected by SD but also open the door for therapeutic interventions and trials. Insights gained from our study could help this situation. There may not be a consensus whether every pwMS should be asked about their sexuality. Yet, especially in times of limited resources, it is helpful to offer patients with particularly high risk for SD methods for screening and counseling (12).

In conclusion, the risk for SD is growing substantially with increasing EDSS but is not associated with depression or anxiety in multivariate analyses. We suggest that screening for SD is particularly relevant for patients with EDSS ≥4.

## Data Availability Statement

The raw data supporting the conclusions of this article will be made available by the authors, without undue reservation.

## Ethics Statement

The studies involving human participants were reviewed and approved by Ethics review board Medical University of Vienna, Austria. The patients/participants provided their written informed consent to participate in this study.

## Author Contributions

PA, FL, KL, and PR contributed to the study concept and design. PA, FL, PR, TM, MP, MS, TZ, and GZ contributed to acquisition of data. PA, FL, GB, MP, MS, and KB helped with analysis and interpretation of data. PA and GB wrote the manuscript. FL, KL, GB, and TB edited the manuscript. PA, MP, and GB had full access to the data in this study and take responsibility for the integrity of the data and accuracy of the analysis. All authors read and approved the manuscript.

## Conflict of Interest

PA has participated in meetings sponsored by and received speaker honoraria or travel funding from Biogen, Merck, Roche, Sanofi-Genzyme, and Teva and received honoraria for consulting from Biogen. He received a research grant from Quanterix International and was awarded a combined sponsorship from Biogen, Merck, Roche, Sanofi-Genzyme, and Teva for a clinical study. FL has participated in meetings sponsored by or received honoraria for acting as an advisor/speaker for Bayer, Biogen, Celgene, MedDay, Merck, Novartis, Roche, Sanofi-Genzyme, and Teva. TM has participated in meetings sponsored by or received travel funding from Biogen, Celgene, Merck, Novartis, Roche, Sanofi-Genzyme, and Teva. PR has received honoraria for consultancy/speaking from AbbVie, Alexion, Almirall, Biogen, Merck, Novartis, Roche, Sandoz, and Sanofi-Genzyme and has received research grants from Amicus, Biogen, Merck, and Roche. TZ has participated in meetings sponsored by or received travel funding from Biogen, Merck, Novartis, Roche, Sanofi-Genzyme, and Teva. KB has participated in meetings sponsored by and received travel funding from Roche. TB has participated in meetings sponsored by and received honoraria (lectures, advisory boards, consultations) from pharmaceutical companies marketing treatments for MS: Allergan, Almirall, Bayer, Biogen, Biologix, Bionorica, Celgene, MedDay, Merck, Novartis, Octapharma, Roche, Sanofi-Genzyme, Teva, and TG Pharmaceuticals. His institution has received financial support in the past 12 months by unrestricted research grants (Biogen, Merck, Novartis, Sanofi-Genzyme, and Teva and for participation in clinical trials in multiple sclerosis sponsored by Alexion, Biogen, Merck, Novartis, Octapharma, Roche, Sanofi-Genzyme, and Teva. GB has participated in meetings sponsored by and received speaker honoraria or travel funding from Biogen, Celgene, Merck, Novartis, Sanofi-Genzyme, and Teva and received honoraria for consulting Biogen, Roche, and Teva. The remaining authors declare that the research was conducted in the absence of any commercial or financial relationships that could be construed as a potential conflict of interest.
